# Supplementary material for: The Molecular Networks of microRNAs and Their Targets in the Drug Resistance of Colon Carcinoma
Source: Cancers (Basel). 2021 Aug 28;13(17):4355. doi: 10.3390/cancers13174355 (PMC8431668; doi:10.3390/cancers13174355)
Supplement: Supplementary file 1 [file cancers-13-04355-s001.zip › cancers-1318929-supplementary/cancers-1318929 - to xml supplementary.pdf]

cancers

MDPI

Supplementary Materials

The Molecular Networks of MicroRNAs and Their Targets in the Drug Resistance of Colon Carcinoma

Francesca Crudele, Nicoletta Bianchi, Annalisa Astolfi, Silvia Grassilli, Federica Brugnoli, Anna Terrazzan, Valeria Bertagnolo, Massimo Negrini, Antonio Frassoldati, and Stefano Volinia

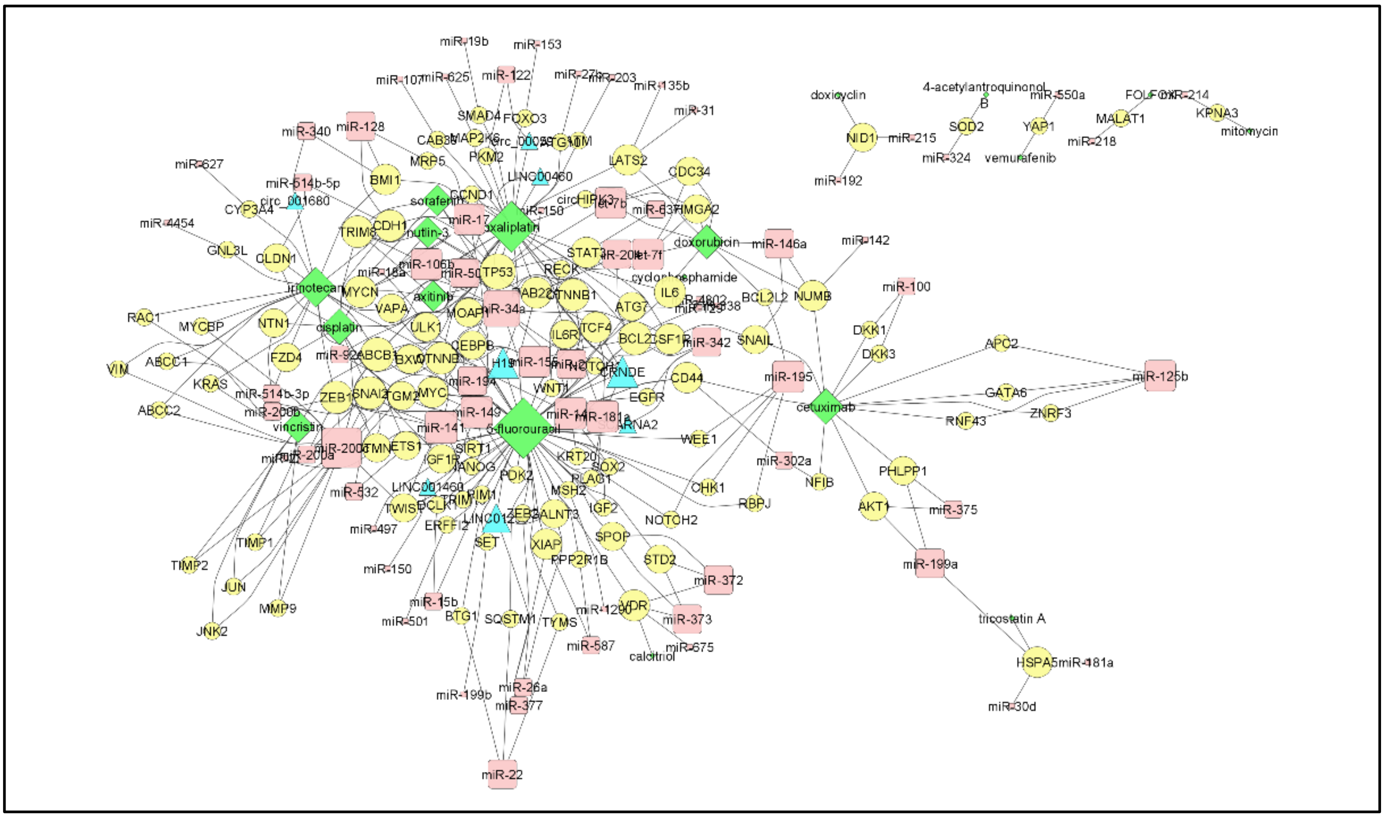

Figure S1. Network of miRNAs and their targets connected to the drugs discussed in our review. In the network we included miRNAs (red rectangle), their target (yellow circle) and miRNA regulators (sky-blue triangle) connected to the drug resistances. The map node size was dependent to degree.

Publisher's Note: MDPI stays neutral with regard to jurisdictional claims in published maps and institutional affiliations.

Copyright: © 2021 by the authors. Submitted for possible open access publication under the terms and conditions of the Creative Commons Attribution (CC BY) license (http://creativecommons.org/licenses/by/4.0/).
